# Handling Neighbor Discovery and Rendezvous Consistency with Weighted Quorum-Based Approach

**DOI:** 10.3390/s150922364

**Published:** 2015-09-03

**Authors:** Chung-Ming Own, Zhaopeng Meng, Kehan Liu

**Affiliations:** 1 School of Computer Software, Tianjin University, Tianjin 300072, China; E-Mails: chungming.own@tju.edu.cn (C.-M.O.); kehanliu@tju.edu.cn (K.L.); 2 Tianjin University of Traditional Chinese Medicine, Tianjin 300193, China

**Keywords:** quorum graph, quorum system, sensor network

## Abstract

Neighbor discovery and the power of sensors play an important role in the formation of Wireless Sensor Networks (WSNs) and mobile networks. Many asynchronous protocols based on wake-up time scheduling have been proposed to enable neighbor discovery among neighboring nodes for the energy saving, especially in the difficulty of clock synchronization. However, existing researches are divided two parts with the neighbor-discovery methods, one is the quorum-based protocols and the other is co-primality based protocols. Their distinction is on the arrangements of time slots, the former uses the quorums in the matrix, the latter adopts the numerical analysis. In our study, we propose the weighted heuristic quorum system (WQS), which is based on the quorum algorithm to eliminate redundant paths of active slots. We demonstrate the specification of our system: fewer active slots are required, the referring rate is balanced, and remaining power is considered particularly when a device maintains rendezvous with discovered neighbors. The evaluation results showed that our proposed method can effectively reschedule the active slots and save the computing time of the network system.

## 1. Introduction

Wireless sensor networks (WSNs) have recently received increased attention for a broad array of applications such as surveillance, environment monitoring, medical diagnostics, and industrial control. A WSN typically includes many inexpensive wireless sensor nodes, each of which can collect, process, and store environmental information. Nodes in WSNs generally rely on portable power sources, such as batteries, to provide the necessary power. Power management of WSNs is critical. Numerous protocols have been proposed to extend the lifetime of sensor networks in their optimizing deployment methods [1,2]. For wakeup mechanisms of pending transmissions, the major objective is to maintain network connectivity when reducing idle-state energy consumption.

Existing wakeup mechanisms include three approaches: on-demand wakeup, scheduled rendezvous wakeup, and asynchronous wakeup. In an on-demand wakeup approach, when nodes receive out-of-band signals, which are used to help the signals or nodes stay on the paging channel, it means that these sleeping nodes wake up in an on-demand manner. However, despite the efficiency of this strategy, complexity and network size dependability are drawbacks [3]. In the scheduled rendezvous wakeup mechanism, system controllers schedule the same wakeup time periodically to communicate with low-power sleeping nodes, such as S-Mac or multi-parent scheme protocols [4]. This mechanism is used in IEEE 802.11 and Bluetooth, and all the involved nodes must synchronize their clocks according to the common wakeup time schedule, in order to exchange their data in time. The drawbacks of this approach are system confirmation and clock synchronization. The final approach is asynchronous wakeup, in contrast to the scheduled rendezvous wakeup approach, which requires no clock synchronization. In other words, each node follows its own wakeup schedule in the idle state and communicates with other overlapping neighbors. To meet this requirement, nodes in this mechanism generally must wake up more frequently compared with scheduled rendezvous mechanisms. Asynchronous wakeup is easier to implement and can maintain network connections even in a highly dynamic situation. However, energy consumption and the robustness of network connectivity trade off in asynchronous wakeup mechanisms [5,6].

Most existing solutions to these types of problems entail using pattern wakeup schedules to satisfy the following requirements:

(a) Discovering the neighbor in a reasonable time frame;
(b) Minimizing the time slots in the node’s wakeup time;
(c) Matching the nodes awake-sleep schedules within their heterogeneous battery duty cycle.

These solutions can be categorized into quorum-based and co-primality based protocols. Quorum-based protocols select wakeup times according to the quorums arranged by the radios’ time slots and add them into the matrix. The row and column arrangements are based on the protocol scheme, which typically assigns active or sleeping slots. Active time slots then overlap with the active time slots of neighboring nodes. When two nodes have the same duty cycle, they can meet each other during the active times from the row and column in the matrix regardless of clock drifts [7]. In addition, co-primality based protocols exploit Chinese Remainder Theorem properties to ensure the same active times in the same slots between two nodes [8]. According to these protocols, nodes wake up at specific time slots multiplied by the protocol parameters, and these numbers are coprime to one another. Thus, nodes can discover one another within a bounded time and delay [9]. In [10], the authors consider the characteristics of sensor network environment, and proposed the mechanism to create exchange keys on the derivation of a one-time password in a two-dimensional quorum system.

This study focused on the development of a dynamic quorum system, which attempts to archive efficient neighbor discovery and rendezvous maintenance. The proposed concept does not follow the two aforementioned solutions; by contrast, nodes can leverage the information of each other nodes. Thus, the neighbor unknown to some nodes can be connected/communicated with assistance from other nodes, such that fewer active slots are possible, and reduced reachability can save node energy. Some studies have regarded rendezvous maintenance as a rediscovery problem, despite the system being scheduled for active slots. To address these two concerns, Zhang *et al.* proposed an extended quorum system for indirect neighborhood information to bridge the multiple pairwise communications, which can avoid the otherwise required full-mesh pairwise connection [11]. They proposed a quorum graph to prove the reachability of the neighborhood information flowing among nodes, and characterized the reduced quorum algorithm to eliminate redundant paths of active slots. However, some drawbacks remain in their methods, including a lack of mobile flexibility and power insufficiency:

- The method proposed by Zhang *et al.*, cannot be applied to mobile dynamic environments; that is, the schedule of a system must be recomputed repeatedly when new devices move around the environment.
- Because redundant reachability leads to energy waste, the network lifetime must be referred to as an additional consideration; however, this concept was not included in the Zhang *et al.*, method.

Thus, a novel method must start from a minimal-delay scheduling solution and subsequently performs energy reduction by applying the power and frequency scaling technique [12]. Especially in the mobile sensor network environment, the limitation of the embedded devices is on their pair reachability and the power maintenances. These are the temporal and spatial considerations in the moving device environment. The reachability ability concerns the spatial usages, and the power usage controls the temporal issues. Hence, to address the quorum reachability problem and to improve the two aforementioned drawbacks in the method proposed by Zhang *et al.*, we designed a heuristic weighted quorum system (WQS) method. This method not only reduces the redundant pairwise reachability between discovered and undiscovered neighbors but also supports the node rendezvous maintenance with a decreased number of active slots to save system energy and eliminate redundant slots.

The remainder of the paper is organized as follows: Section 2 presents the theoretical concepts for quorum graphs and an extended quorum system. Section 3 formalizes the quorum reachability minimization problem, followed by an evaluation of the proposed WQS algorithm in Section 4 and Section 5 discusses related works and concludes the paper.

## 2. Literature Review

In this section, we introduce the theoretical concepts of neighbor discovery and rendezvous maintenance. In addition, we discuss the EQS method proposed by Zhang *et al.* [11].

### 2.1. Wakeup Schedule

**Figure 1:**
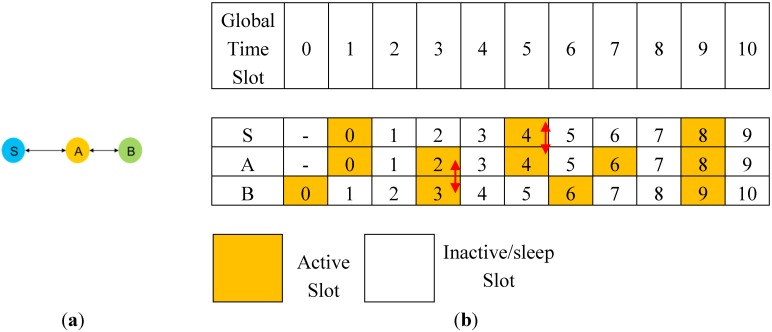
(**a**) Example of rendezvous maintenance; (**b**) example of neighbor discovery and rendezvous maintenance.

This study considered a time-slotted WSN in which each node is energy-constrained. Neighbor discovery among nodes allows two nodes with independent duty cycles and no prior synchronization information to discover each other periodically when the nodes are within their radio range. A rendezvous allows nodes to deliver messages to previously discovered neighbors with predictable latencies. Nodes follow a neighbor discovery wakeup schedule that defines the time pattern required to wake up or sleep, so that nodes in the network can discover their respective neighbors in an energy-efficient manner. Neighboring nodes share common neighbors by using many techniques [13,14]. If nodes broadcast their two-hop neighbor tables in active slots, a node can indirectly discover its new neighbors through a direct rendezvous with assistance from the already discovered neighbor. Figure 1a shows the example of rendezvous maintenance. Node S can contact node B via neighbor node A.

**Figure 2:**
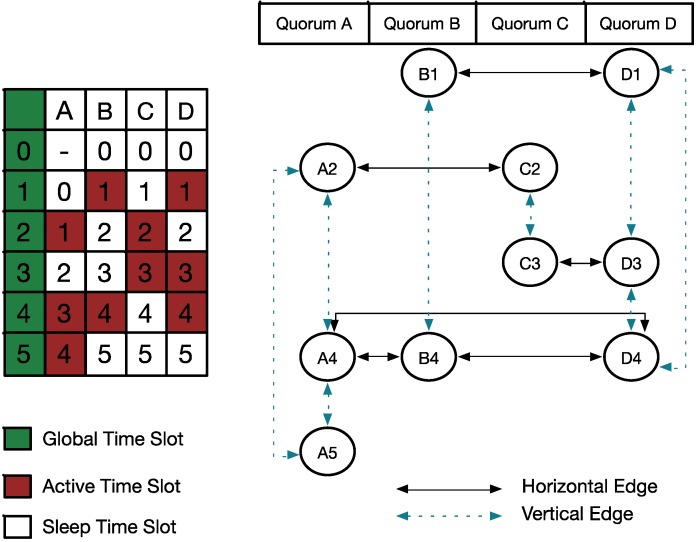
Example of quorum graph.

Node S can rendezvous indirectly with another node B by directly rendezvousing with an intermediate node A. In other words, nodes A and B have already been rendezvoused. This is achieved by node A, which passes the neighbor table of node B to node S. In addition, based on the state-of-the-art discovery protocol DISCO [9], two nodes, A and B, start counting the passage of these periods, which are divided into fixed-width reference periods and consecutive periods labeled with consecutive integers. Figure 1b shows the neighbor discovery and rendezvous maintenance among nodes S, A, and B. Figure 2 shows three nodes S, A, and B. According to the DISCO protocol, node S directly rendezvouses with its already discovered neighbors A and B in global time slots 4 and 8. In addition, node S can continue to indirectly discover the neighbor in global time slots 0, 1, 4, and 8. Before time slot 9, node S lacks the opportunity to connect directly with node B. However, in global time 0, nodes S and A can rendezvous their neighbor table. Node A’s table containing the neighbors of S propagates to B. Therefore, even if node S cannot directly rendezvous with node B before global time 9, node S can rendezvous with B by using a transiting relationship that is the rendezvous between nodes A and B and between S and A in global time 3 and 5, respectively. These transiting relationships, which are denoted by red arrow lines in Figure 2, indicate the indirect connection for the resource saving reasons. Considering neighbor discovery and rendezvous maintenance in our system design, it is possible to eliminate the need for fully meshed pairwise discovery and rendezvous.

### 2.2. Quorum Graph

According to the preceding discussion [11], the quorum system can be represented as a quorum graph, which is denoted as *G*(*V*,*E*). The quorum graph comprises a vertex, a subgraph, a supergraph, and edges.

(1) Vertex: Vertices can be used to represent active slots for each node.
(2) Subgraph: For a quorum, all vertices to one node can be represented as a subgraph.
(3) Supergraph: Subgraphs corresponding to all the nodes can be represented as a supergraph.
(4) Edge: Two vertices correspond to the same active slot in two subgraphs. A horizontal edge is used to connect these two vertices, which indicates that two nodes in this slot can exchange neighborhood information bidirectionally. Conversely, if two nodes corresponding to the two active slots in the same subgraph can pass neighborhood information only unidirectionally, a unidirectional vertical dashed edge exists between these two nodes.

**Figure 3:**
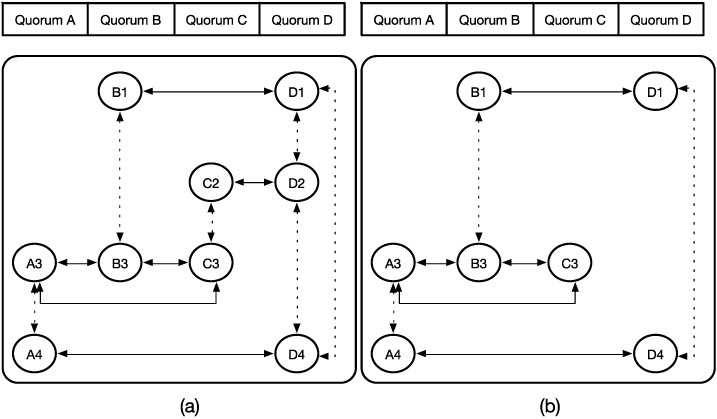
Example of (**a**) legacy quorum graph; and (**b**) extended quorum graph.

Figure 2 illustrates the construction of a quorum graph based on a network schedule. The network consists of nodes A, B, C, and D, as shown on the left side of the figure, and the global time slots range from 0 to 5; each node has its own wakeup schedule denoted by a red brick. Hence, according to the previous definition, we designed the quorum graph, as shown on the right side of Figure 3. This figure has four subgraphs (quorums A, B, C, and D), and the vertices represent active time slots in the network schedule. In addition, the horizontal dashed edges, that is, A2, A4, and A5 from the early to later slots within the subgraph, can be used to pass neighbor information. Vertical solid edges, for example, A4, B4, and D4, are built for the vertices in different subgraphs but associated with the same slot; these three nodes are associated with the same (global) time slot 4.

### 2.3. Extended Quorum Graph

In [11], the authors proposed an extended quorum system for neighbor discovery and rendezvous. The system is based on an improvement of the legacy quorum system. In the legacy quorum system, quorum information is exchanged directly without the involvement of intermediate quorums, and only the horizontal solid edges are used in the quorum graph. The extended quorum system employs vertical dashed edges to reach other nodes indirectly. Thus, the indirect connection can increase the efficiency of information transfer between nodes. Figure 3 shows a legacy quorum graph and extended quorum graph. In Figure 3a, because of the direct connection, all of the nodes can exchange information in the four active time slots. However, with the intermediate quorum involved, as shown in Figure 3b, time slot 2 can be reduced to save energy.

## 3. System Framework

**Figure 4:**
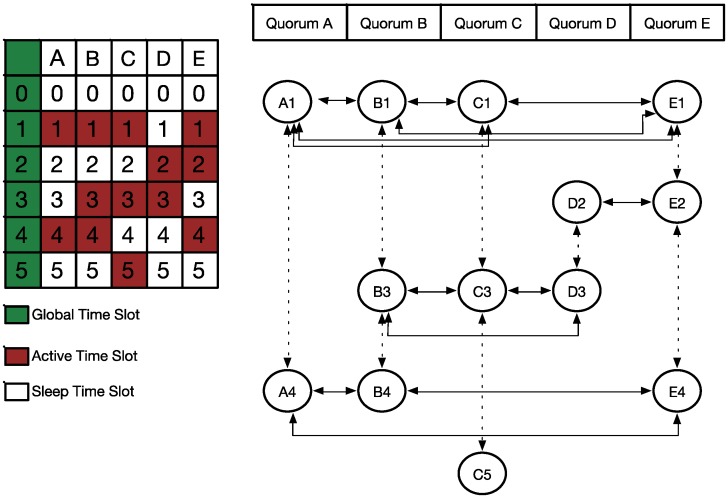
Example of our proposed WQS method.

According to its definition, a legacy quorum system ensures that all the nodes in a network can successfully and directly discover and rendezvous with other nodes, and the system focuses on only pairwise and direct discovery and rendezvous. Although implementing it is extremely easy, the energy consumption for each node is high. However, through the proposed extended quorum system, the concept of indirect reachability can be used to discover and rendezvous through already discovered neighbors. For the example in Figure 4b, the information of node B can be indirectly propagated to node D through the intermediate node A. Thus, the redundant active time slots can be eliminated. However, the extended quorum graph does not completely support the mobile environment, especially in the flexible network design and device energy consideration.

Mobile devices are represented as mobile nodes in a mobile environment, and they constantly reconfigure. The schedule of active time slots should be modified for the nodes that join or leave, when the device owners enter or leave the area. However, according to the extended quorum graph, the node system takes time to rearrange the active time slots when the node structure is changed. In addition, the battery life for each node is different. To extend the network life and strengthen the network performance, the proposed method should consider the battery usage. Thus, we designed and proposed our Weighted Quorum method (WQS).

The main concept of the proposed WQS method is simple and based on the following observations: (1) flexibility: the optimizing arrangement of active time slots; (2) mobility: in a mobile environment, configuring schedules for the new nodes in the network is easy; and (3) energy efficiency: the consideration of the nodes’ energy state in the active time slot arrangement.

In our developing idea, we try to maintain the most connections per row in the quorum graph, and the redundant connections would be deleted to increase the energy efficiency. For maximal contribution to pairwise connections in our study, we defined the decision factor in Equation (1) for a vertex $x_{i}$ in each row, $f_{x_{i}}$ , is used as the idea to select the needed pairwise connection. Thus,

(1) $$\begin{array}{c} f\left(x_{i}\right)=\left(1-\alpha \right)b_{x_{i}}+\alpha a_{x_{i}}\\ f_{y}=\;\left(1\;-\;\alpha \right)b_{y}+\;\alpha a_{y} \end{array}$$

where $b_{x_{i}}$ is the node energy status, and the accumulative wakeup factor is $a_{x_{i}}$ , which is the reciprocal of the connecting numbers. The energy status is the ratio of the remaining power for each node. The decision factor $f\left(x_{i}\right)$ is used to decide the candidates among the selected vertices. The α depends on the system requirement. Because the proposed system is used in a mobile environment, the energy status could be distinct for each node in the environment. The accumulative wakeup factor is the reference status for each node. The more the node was referenced, the lower the reference status is than those of the other nodes. In our experiment, α is defined as 0.5 in our system evaluation. For the purpose to illustrate the importance of reference rate and energy status, we compare the distinct results of α value in the experiment 4.3.

Accordingly, we divide the quorum graph into two parts by the row with the most vertex numbers in one row. In other words, assume that the network has *M* nodes and *N* active slots. Thus, we have $\;X=\left[x_{1},x_{2},\;\ldots ,\;x_{M}\right]\;$ and $A=[A_{1},A_{2},\;\ldots A_{N}]$ , a total of N × (N − 1) connections is retained with a minimal number of active slots in the legacy quorum graph. For the active slot $A_{i}$ , we select the slot with many nodes as $A_{q}$ ,  1≤i,q≤N , Then, then the quorum has been divided, $A=[A_{1},A_{2},\;\ldots A_{N}]=[\bar{A},A_{q},\underline{A}]$ , where $\bar{A}=\left[A_{1},A_{2},\ldots ,A_{q-1}\right]$ and $\underline{A}=\left[A_{q+1},A_{q+2},\ldots A_{N}\right]$ . Then, we scan and try to find the row containing the most complement vertices of $A_{q}$ for each divided part. Every time a row of complement vertices has been chosen, a new connection would be built. Then we perform the filtering processing, these unselected vertices are pick up and contribute to the maximal number of decision factors. This process runs repeatedly until the selected vertices can pair to all the nodes directly or indirectly.

Thus, we scan each row to find the vertices set contained maximum unselected vertices by

(2) $$u=\arg\max_{j}(║{\bar{A}}_{j}-A_{q}║)\;1\leq j\leq q-1,\text{ and }A_{q}\subset {\bar{A}}_{u}$$

or

(3) $$b=\arg\max_{j}(║{\underline{A}}_{j}-A_{q}║)\;q+1\leq j\leq N,\text{ and }A_{q}\subset {\underline{A}}_{b}$$

where *u* and *b* are represented as the selected row with the maximum unselected vertices in the two divided block respectively. These two groups, $A_{q}\;\text{to }A_{u}$ , and $A_{q}\;\text{to}\;A_{b}$ can share the most responsibilities of rendezvous maintenance. Besides, based on the idea of extended quorum graph, nodes can exchange information with the intermediate nodes’ help. Our system only keeps only the one overlapping node in the newly selected row as the bridging node. Thus, we compute the energy status of the overlapping nodes in $\;A_{u}\;\text{and}\;A_{b}$ by Equation (1), and remove the nodes with the less decision factors, which are defined as ${\bar{A}}_{u}\;\text{and}\;{\underline{A}}_{b}$ . We repeat the previous scanning procedure until we connect all the nodes in the upper and lower blocks.

**Figure 5:**
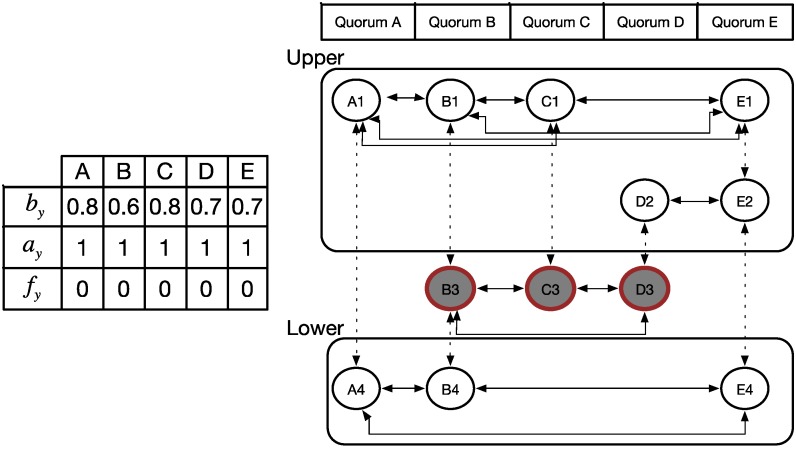
Two parts of WQS method.

**Figure 6:**
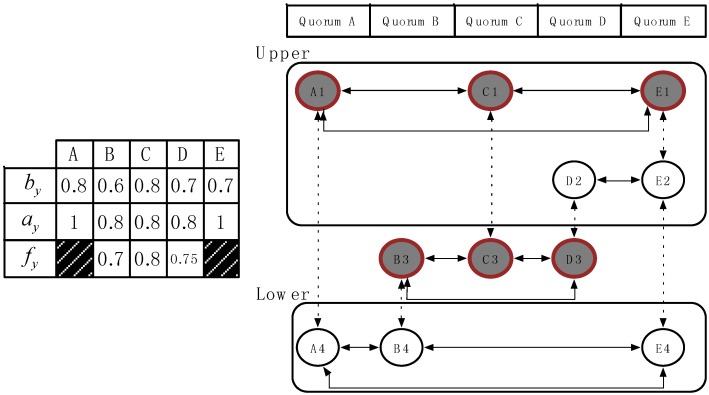
Decision result in the WQS method.

**Figure 7:**
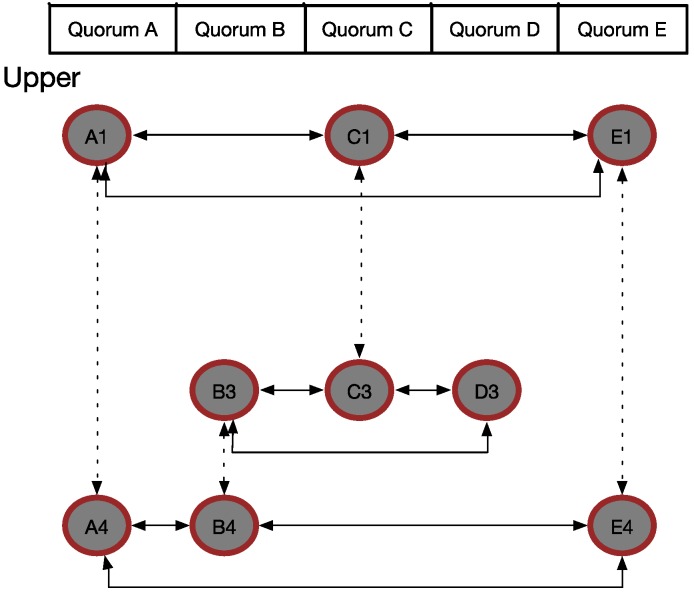
Final result of our example.

Figure 4 shows an example of the proposed WQS method. The network has five nodes, A–E, each of which has its own distinct active slots. Converting to the quorum graph, we have five vertices respectively. The steps performed for WQS are listed as follows:

(1) According to the involved schedule, we derived the corresponding quorum graph. In addition, we assumed the energy status for each node to be 0.8, 0.6, 0.8, 0.7, and 0.7 in this example. Hence, we have *X* = [A, B, C, D, E] and $A=[A_{1},A_{2},\;A_{3},A_{4},\;A_{5}]$ .
(2) For performance reasons, we removed the row with only one vertex. Considering Figure 4, we removed row 5 with vertex C5. Then, $A=[A_{1},A_{2},\;A_{3},A_{4}].$
(3) According to the quorum graph shown in Figure 5, we have *N* global time slots; therefore, we selected the row with the most vertices as our first demanded row. In this example, we assigned row 3 as our demanded row $A_{q}$ . Nodes B, C, and D were our first selecting vertices.
(4) Figure 5 shows the next step. Because row 3 was assigned as the demanded row, the subgraph of the quorum graph was divided into two blocks, upper $\bar{A}$ and lower $\underline{A}$ , by the demanded row $A_{q}$ .
(5) In the upper block $\bar{A}$ , we must select the next demanded row. Because we selected the nodes $S_{x}=\;\left\{\text{B},\text{C},\text{D}\right\}$ in our first demanded row, we obtained the candidate nodes $C_{x}=\;\left\{\text{A},\text{E}\right\}$ , which denote the remaining unselected vertices. Hence, in the upper block, rows 1 and 2 were not selected, and row 1 had more unselected vertices in $C_{x}$ than row 2. Hence, row 1 is our new selected row, that is $A_{u}$ .
(6) Accordingly, we must find the overlapping vertices to connect to the previous demanded rows. Using Equation (1), we can compute the decision factor of the overlapping nodes as {B, C, D}. Hence, the derived results are 0.7, 0.8, and 0.75 for nodes B, C, and D respectively. For the purpose to save the energy or keep the reference rate, we pick the node C with the maximum value as the bridge node, then we remove all the edges between rows 1 and 2, expected the connection to node C. The newly row is ${\bar{A}}_{u}$ . The new result was modified, as shown in Figure 6.
(7) When we added these new selected vertices into the selected vector, the selected nodes became {A, B, C, D, E}, and the candidate vector was empty. The goal of the upper block, which was to connect all the vertices directly or indirectly, was fulfilled.
(8) The lower block can be worked upon in a similar manner; the final result is shown in Figure 7.

## 4. System Evaluation and Analysis

To evaluate the performance of the proposed method, we first conducted a comparative analysis among DISCO [9], EQS [11], and our proposed WQS method. We then performed a series of simulations to evaluate WQS in several typical application scenarios for network lifetime and network reconfigure time. In our simulations, several mobile devices were uniformly deployed in a square area of size 30 m × 30 m. The transmission ranges of the devices were set at 20–110 m, and the average mobile device density was computed from 4 to 60 neighbor devices. Each simulation was repeated 10 times and the average results are listed as follows.

### 4.1. Effect of Network Lifetime on Different Device Densities

**Figure 8:**
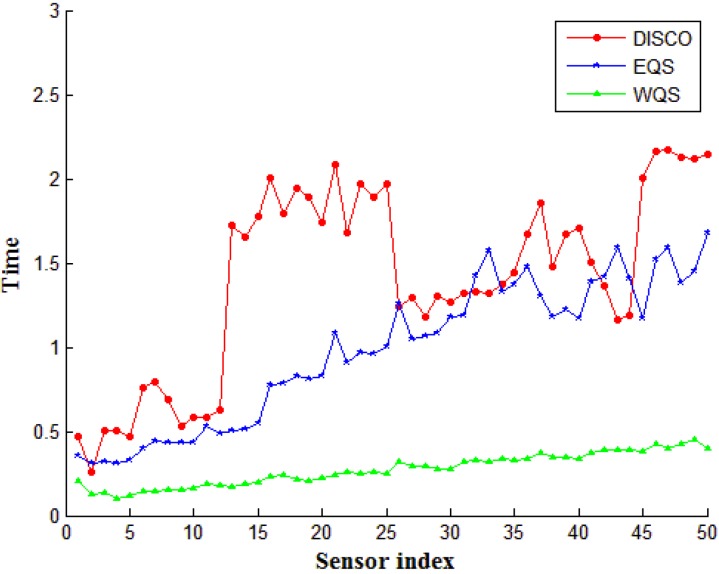
The average duty cycle time on different device densities.

In this simulation, we compared the effective energy conservation from the proposed WQS method with EQS and DISCO for different device densities. The effect of device densities on energy consumption, represented by the average duty cycle time of all devices in the network, is shown in Figure 8. A duty cycle is the percentage of one period in which a signal is active, and a period is the time of system takes for a signal to complete an on-and-off cycle. In Figure 8, the *x*-axis is the sensor index number, and the *y*-axis represents the average time of duty cycles for the sensors. The execution time is from the network starting to all nodes stopping. A shorter duty cycle denotes superior sensor performance. Thus, the performance of WQS is considerably superior to DISCO and EQS. A remarkable difference exists between WQS and EQS, which is based on the selection of the intermediate nodes. Figure 8 shows the difference; when the device density increases, the time of the average duty cycle increases in EQS, because the nodes in EQS cannot balance the communication burdens to the others. Many pairwise connections are concentrated on the same nodes, that will require more wakeup instances to handle the communication. The same situation exists for DISCO. By contrast, the proposed WQS method is based on the balanced selection of intermediate nodes, which can facilitate connecting two rows. When the proposed system attempts to select a pairwise node, the balance condition between the remaining power and referencing time is considered. Hence, more connection burdens can share to those nodes in the network, when the network structure has been changed. In general, the average duty cycle time is decreased from 8.88% and 58.28% than EQS and DISCO respectively.

**Figure 9:**
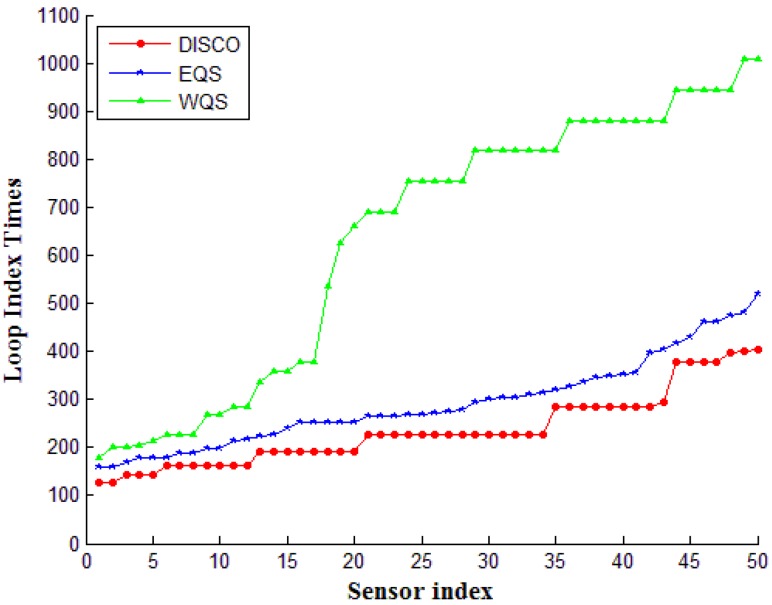
The network lifetime on different device densities.

Accordingly, Figure 9 shows the average times of the loop index for each node in the different network density. In software engineering, a loop index is the variable that counts the iteration of a loop. In this simulation, the numbers of loop index are computed for the comparison of our proposed method and the other two methods, the index numbers are accumulated until energy exhausts the nodes. The bigger index numbers means better performances of scheduling flexibility and resource allocation. In Figure 9, the average times of the loop index for the comparison of DISCO, EQS, and WQS are based on the different network size, from 2 to 50 nodes. Figure 9 shows that the index times of the proposed method is considerably superior to that of the other two methods. That is, for the same job, the loop in our proposed method can execute faster and exchange more information than the other two methods. This advantage happens by the balance condition for each node. Besides, Figure 9 illustrates the WQS method has the advantages on the heavy loading network, the more nodes can share the connection burden and keep the stable increasing times obviously. The robust power can be proven herein. According to our accumulation, in the same execution time, the average loop index times in our proposed method have 69.94% and 78.39% more than the EQS and DISCO methods.

### 4.2. Effect of Network Lifetime on Dynamic Network

**Figure 10:**
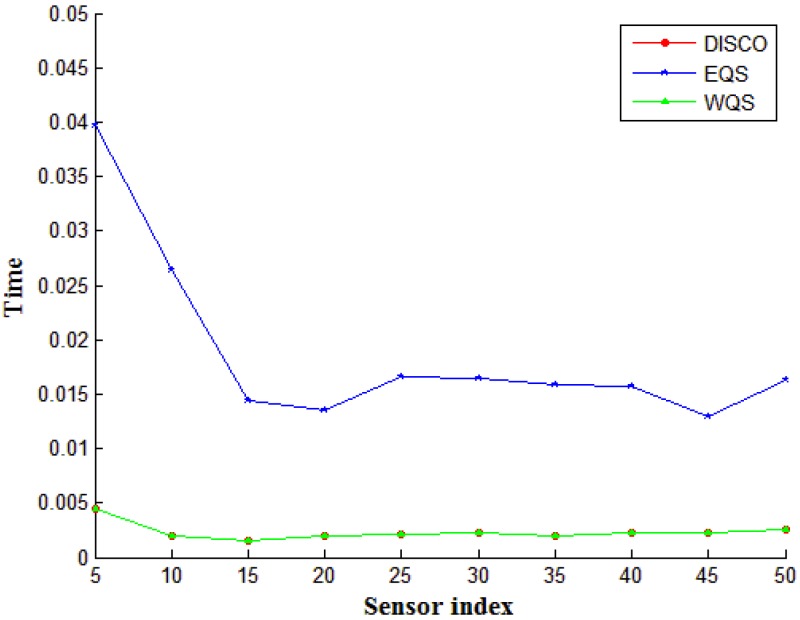
The computation time on different device densities.

In this simulation, we emulated the dynamic mobile network environment; that is, the mobile devices could move randomly throughout our network. We compared the reschedule time of the active slots when a new device joined the network (1–50 nodes in order). The comparison results are shown in Figure 10. According to system definitions, DISCO and our proposed WQS methods can append the newly moving node in the demanded row computed by the first step. Thus the system can reschedule the active slots in a very short time. That is why the system time of DISCO and WQS methods is equalized to zero in Figure 10. By contrast, EQS must reconfigure the slot structures of the entire process again. The computation time is considerably longer than that of the other two methods.

### 4.3. Effect of Decision Factors on Distinct Network Density

**Figure 11:**
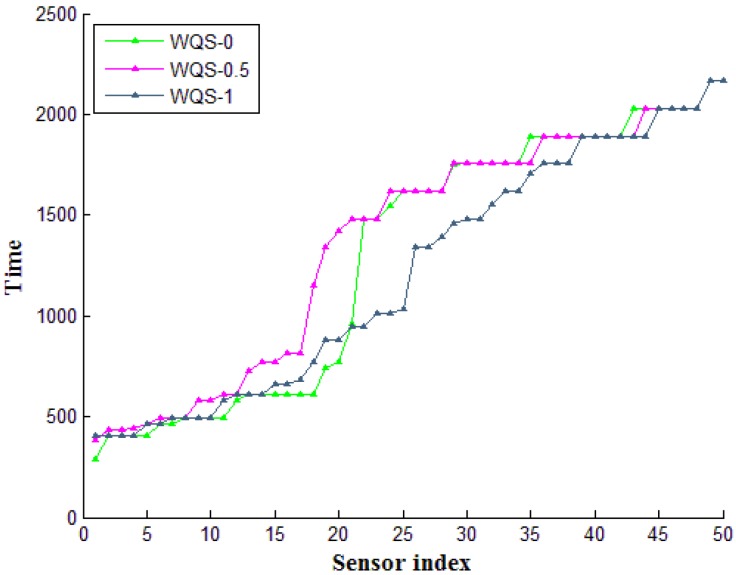
The network time on different sensor density.

According to Equation (1), we try to find the optimum decision factor to maintain the system balance between reference ratio and energy status. Hence, we applied our methods to α= 0, 0.5 and 1 respectively. The system is evaluated with the distinct network density (from 0 to 50), and the network times are gathered in Figure 11. In Figure 11, we collect all the network executing time till the network shutdown. According to the data, we can distinguish that the decision factor with α= 0 and 0.5 retained the longer network execution time. On the contrary, the decision factor with α= 1 is with the less performance. Hence, the system balance should be addressed with the consideration with reference ratio and energy status. When the network density is small, the factor with energy status is more important than the reference ratio. Furthermore, when the network density increases, the system should focus on the reference ratio to retain the structure’s balance, instead of the energy status. The intermediate nodes could be wasting energy when the system counts to the same nodes to connect with other nodes. Thus, in our system, we all adopted α= 0.5 as the optimum value.

## 5. Conclusions

This paper proposed WQS, a decision procedure for balancing the energy conservation and referring ratio for existing neighbor discovery and rendezvous maintenance schemes for a pairwise connection. We mainly focused on the insight that a node can share its neighbors in the same active slot, and can indirectly leverage the knowledge to its unknown neighbors. In other words, fewer active slots are required, the referring rate is balanced, and remaining power is considered particularly when a device must maintain rendezvous with previously discovered neighbors. We improved the extended quorum system and proposed a graph reduction algorithm, WQS, for balancing the referring rate and remaining power for each node. We evaluated the system performance by using DISCO, EQS, and WQS. The evaluation results showed that the proposed method can effectively reschedule the active slots and save the computing time of the system.
